# Complete Genome Sequence of a Field Strain of Peste des Petits Ruminants Virus Isolated during 2010-2014 Epidemics in Senegal

**DOI:** 10.1128/genomeA.00772-14

**Published:** 2014-09-25

**Authors:** Habib Salami, Guillaume Croville, Olivier Kwiatek, Jérôme Mariette, Christophe Klopp, Sophie Valière, Jean-Luc Guérin, Moustapha Lo, Yaya Thiongane, Emmanuel Albina, Geneviève Libeau

**Affiliations:** a CIRAD, UMR CMAEE, Montpellier, France; b INRA, UMR 1309 CMAEE, Montpellier, France; c Université de Toulouse, INP, ENVT, Toulouse, France; d INRA, UMR 1225 IHAP, Toulouse, France; e Plateforme Bioinformatique Toulouse, Midi-Pyrénées, UBIA, INRA, Auzeville Castanet-Tolosan, France; f INRA, UMR 444 Laboratoire de Génétique Cellulaire, INRA, Auzeville, Castanet-Tolosan, France; g GeT-PlaGe, Genotoul, INRA Auzeville, Castanet-Tolosan, France; h Institut Sénégalais de Recherches Agricoles, LNERV, Dakar-Hann, Senegal; i CIRAD, UMR CMAEE, Petit-Bourg, Guadeloupe, France

## Abstract

Peste des petits ruminants virus (PPRV) infection is expanding and results in regular epizootic activities in Africa, the Middle East, and Asia. Here, we report the complete genome sequence of a field strain of PPRV isolated in Senegal (SnDk11I13) in 2013.

## GENOME ANNOUNCEMENT

The recurrent emergence and spread of peste des petits ruminants virus (PPRV) in recent years have been associated with high losses of goat and sheep livestock. In addition to the highly contagious nature of the disease, the rapid spread is attributed to a combination of factors, including human population growth, increased trade, and exchanges of animals and limited implementation of control measures against the disease. The huge health and economic impacts of the disease and the existence of a highly effective vaccine have resulted in recognizing PPRV as the next animal disease to be eradicated after rinderpest.

Analyses of pathological samples collected during different PPR outbreaks in Senegal since 2010 have disclosed the prominent spread of lineage II instead of lineage I, which was formerly the dominant, if not the unique, lineage found in West Africa (1–3). The PPRV genome described here is derived from direct analysis of an eye swab sampled from a goat exhibiting typical clinical symptoms. PPRV whole RNA was extracted from the expurgated swab by using the Viral DNA/RNA isolation kit (Macherey-Nagel) and then processed for next generation sequencing. In brief, first and second strand synthesis and random PCR amplification were performed. PCR products were purified and processed for high throughput sequencing with a MiSeq sequencer (Illumina, San Diego, CA) using a paired-end read length of 2×300 nucleotides (nt) with the Illumina MiSeq reagent Kits v3 (Illumina).

After bioinformatics analysis, the remaining nucleotides gaps (4.72% of the genome) localized in N, M, F, and L were filled using conventional reverse transcription and sequenced using specific primers (Cogenics, United Kingdom). The nucleotide sequences of the 3′ leader region were determined by rapid amplification of cDNA ends (RACE) (Roche) using specific primers binding in the leader sequence and poly(A) tailing using terminal transferase (TdT) and dATP. Then, the completed genome was realigned against the reference genome (X74443).

Genotyping, according to a previous publication (4) using the 5′ end of the N gene, classified the present field strain into lineage II (out of the four PPRV lineages). The genome organization of the strain SnDk11I13 is consistent with previously published sequences for PPRV, with a size of 15,948 nt, fulfilling the “rule of six” and a gene order 3′-N–P/V/C-M-F–H-L-5′. It shares high identity (99.2 to 99.9%) on the complete N gene with other strains sampled in various locations in Senegal during 2010 to 2014. All these strains without exception belong to the same lineage II.

There are currently 11 full genomes of PPRV available, including two vaccine strains, but only 1 is from lineage II (Nigeria 1976 strain). We believe that the availability of more whole-genome sequences as well as relevant epidemiological data related to sampled animals will provide the discriminatory skills needed to establish the diversity of field strains and facilitate source tracking.

### Nucleotide sequence accession number.

The complete genome sequence of the SnDk11I13 PPRV strain is available at GenBank under the accession no. KM212177.
